# Supplementary material for: Social patterns underlying a new group formation in olive baboons
Source: PLoS One. 2025 Oct 22;20(10):e0333541. doi: 10.1371/journal.pone.0333541 (PMC12543123; doi:10.1371/journal.pone.0333541)
Supplement: S1 Appendix — Additional figures and tables detailing our causal assumptions, posterior estimates from our statistical models, and model outputs. (DOCX) [file pone.0333541.s001.docx]

**Figure S1.** Directed acyclic graphs depicting our assumptions regarding the relationships between our predictor variables for (a) female-female relationships and (b) female-male relationships, produced using DAGitty (https://www.dagitty.net/). In (a), we expected current group membership to be underpinned by maternal relatedness, given the prior history of fission that led to the creation of ENK. Current rank and maternal relatedness may also shape both eventual outcomes and grooming relationships, while current co-residence constrains eventual outcomes (i.e., individuals in the same group are likely to stay together). In (b), we expected male rank to shape eventual outcomes, grooming relationships, and siring histories, as high ranking males achieve more mating opportunities. Likewise, we expected siring history to shape both eventual outcomes and grooming relationships and to be impacted by co-residence, as co-resident dyads are more likely to have shared offspring. This schema may seem counterintuitive, as grooming behavior preceded these eventual group membership outcomes. However, we use eventual outcome here to capture the baboons’ latent social preferences, which are reflected both in their current group membership (as females chose to stay in the same group during previous fissions) and eventual outcome (as females are calibrating their social decisions). Green variables indicate exposures of interest, blue variables indicate outcomes, and white variables indicate adjustment or “control” variables. Gray variables are latent and unmeasurable but indirectly captured through the inclusion of exposures and adjustments.

**
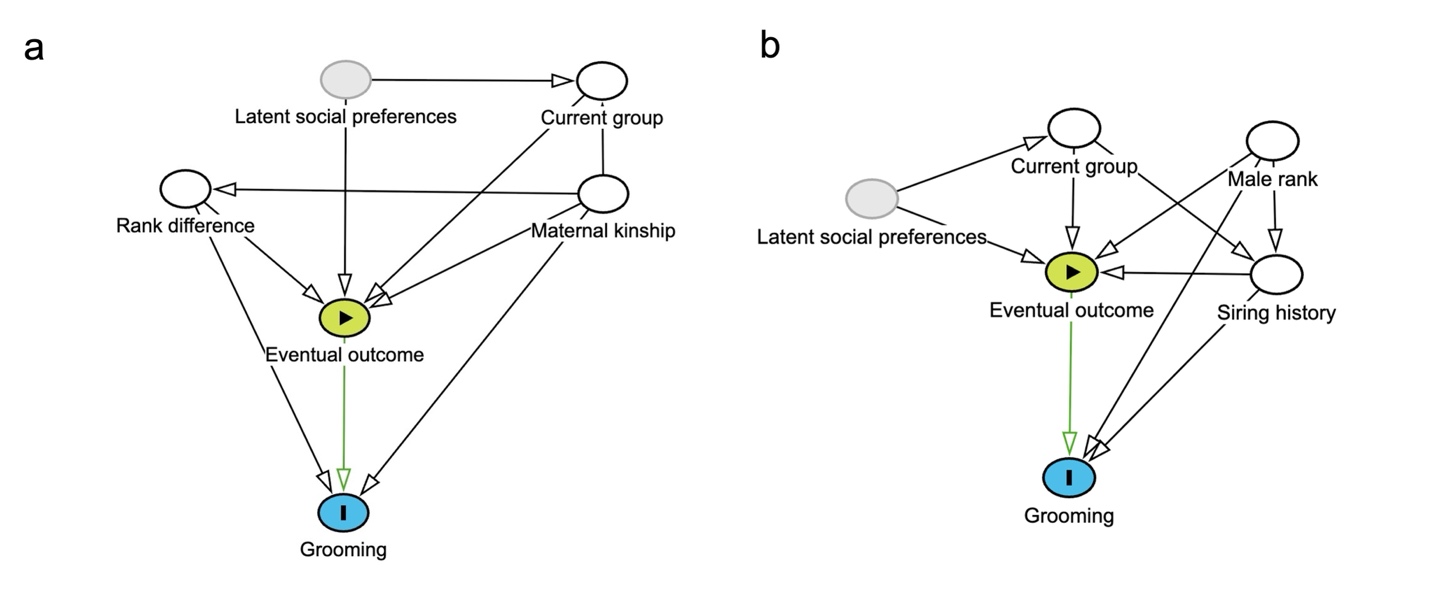
**

**Figure S2. Posterior distributions of parameter estimates from (a) Model 1c (female-female dyads) and (b) Model 1f (female-male dyads).** Light blue shaded areas indicate 89% credible intervals, and dark blue lines indicate posterior means. Labels ending in ‘z’ indicate parameters associated with the model’s hurdle component, while labels ending in ‘g’ indicate parameters associated with the model’s gamma component.
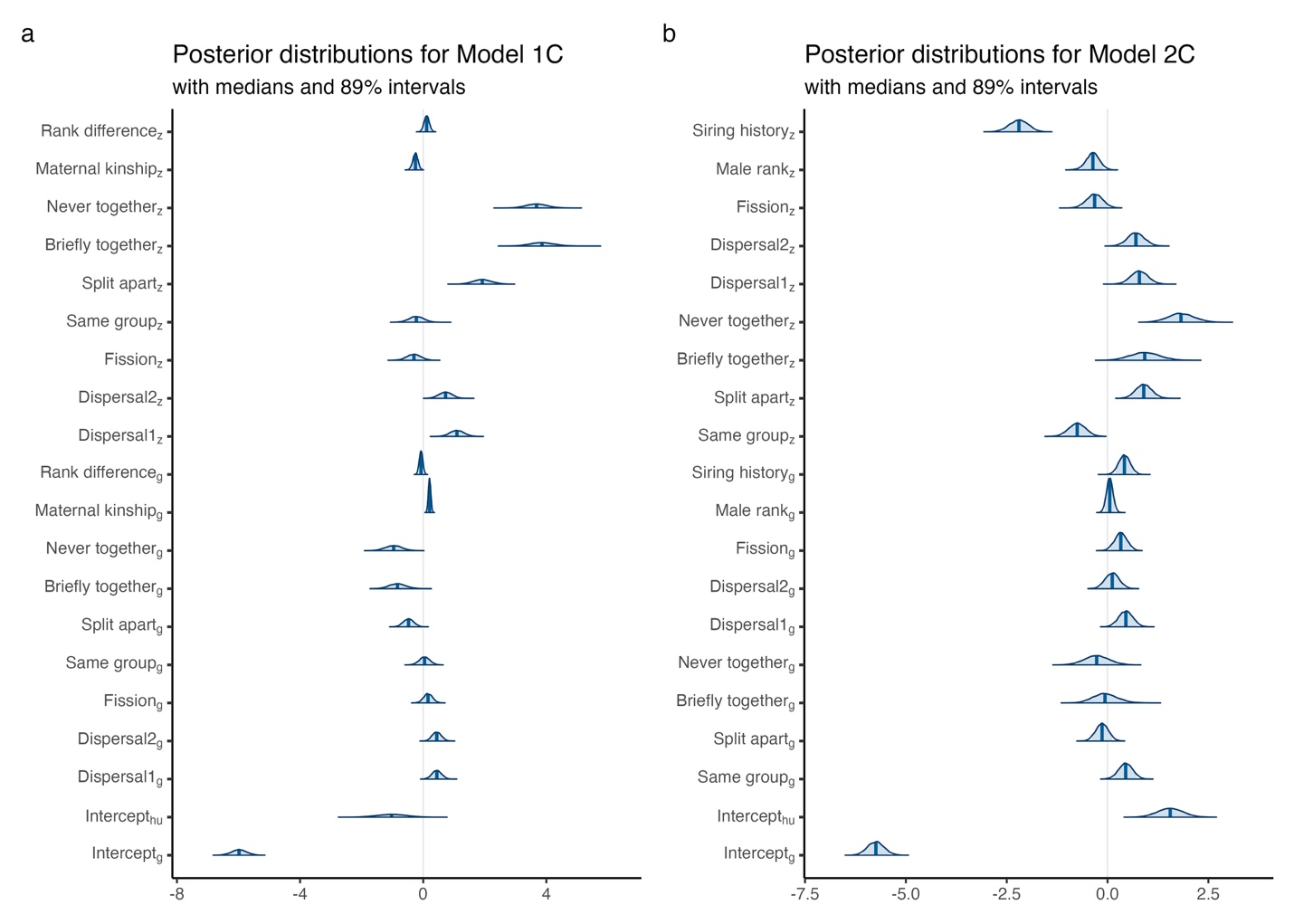


**Figure S3. Posterior distributions of parameter estimates from (a) Model 3A (individual-level aggression), (b) Model 3B (dyad-level aggression), (c) Model 3C (aggression intensity), and (d) Model 3D (intergroup aggression outcomes).** Light blue shaded areas indicate 89% credible intervals, and dark blue lines indicate posterior means.


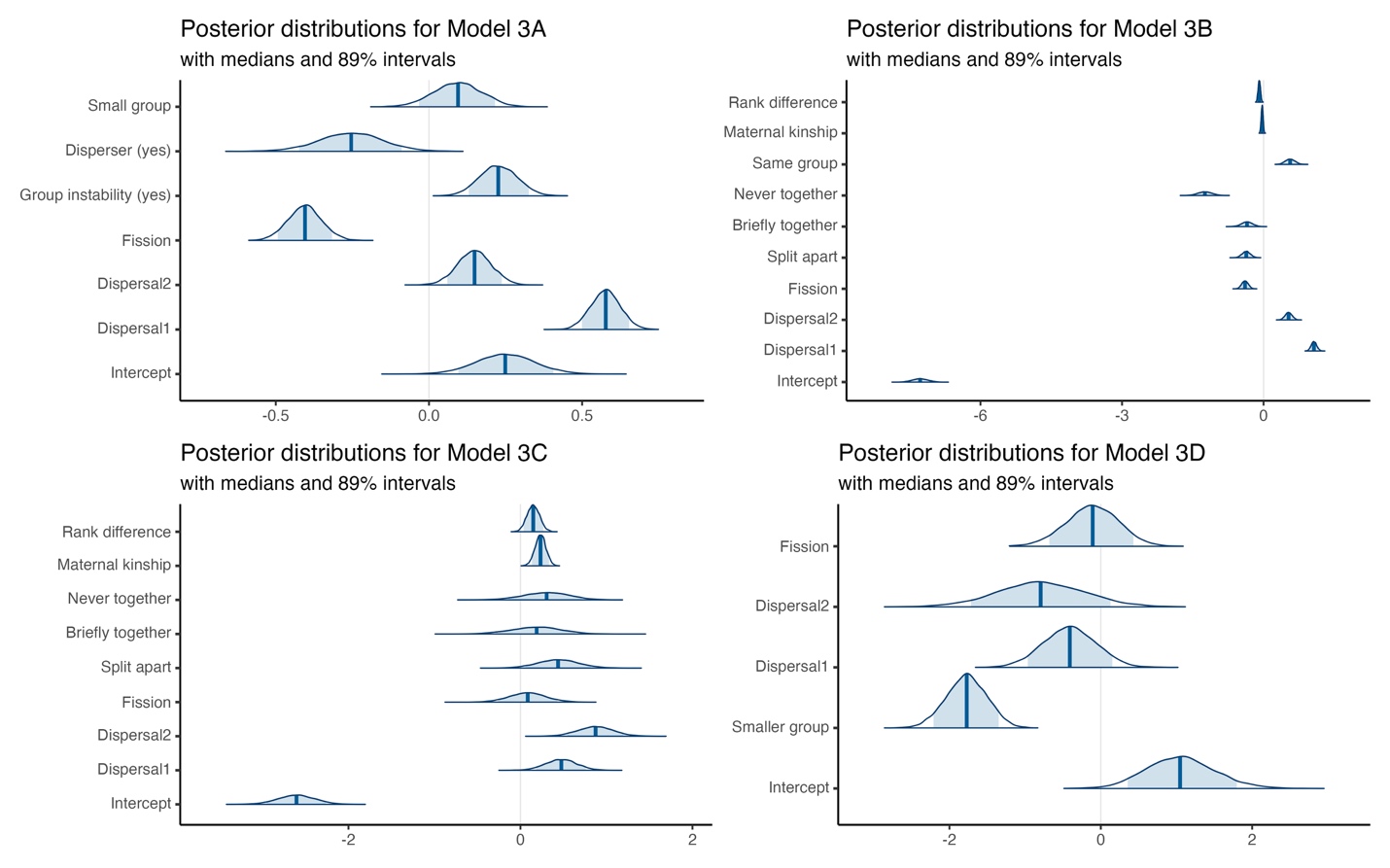


**Figure S4. Posterior distributions of parameter estimates from Model 4, which focused on intergroup grooming patterns**. Light blue shaded areas indicate 89% credible intervals, and dark blue lines indicate posterior means.

**
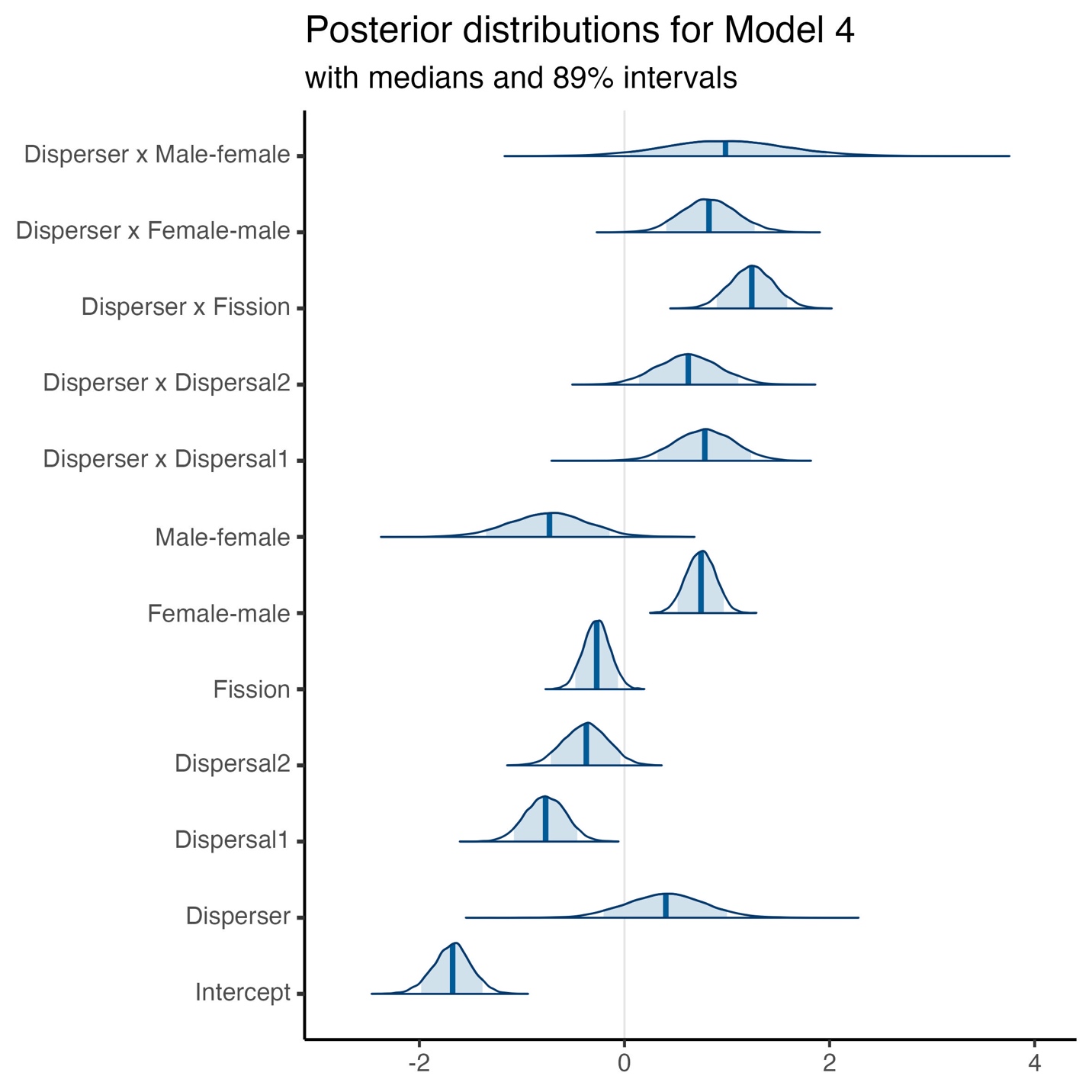
**

**Table S1. Parameter estimates from the model examining how co-residence shaped female-female grooming relationships (Model 1a).** Results from a ‘hurdle gamma’ model examining the impacts of co-residence, sampling period, relatedness, and rank on female-female grooming relationships (n = 1521 dyad-years). β_z_ indicates parameters for the hurdle component (where positive values indicate a decreased likelihood of a zero outcome), while β_g_ indicates estimates from the gamma portion of the model (where positive values indicate an increased duration of grooming).

| **Parameter** | **Estimate** | **Std. Error** | **89% CI LL** | **89% CI UL** | **Rhat** | **Bulk ESS** | **Tail ESS** |
| --- | --- | --- | --- | --- | --- | --- | --- |
| Intercept_z_ | 1.94 | 0.33 | 1.41 | 2.47 | 1.00 | 2101 | 3479 |
| Intercept_g_ | -6.55 | 0.19 | -6.86 | -6.26 | 1.00 | 3616 | 4513 |
| **β_z_ Co-residence (Same group)** | **-2.19** | **0.15** | **-2.43** | **-1.95** | **1.00** | **9767** | **5595** |
| **β_g_ Co-residence (Same group)** | **0.50** | **0.12** | **0.30** | **0.69** | **1.00** | **8716** | **6460** |
| **β_z_ Period (Dispersal 1)** | **0.79** | **0.20** | **0.46** | **1.11** | **1.00** | **7248** | **6193** |
| **β_g_ Period (Dispersal 1)** | **0.44** | **0.15** | **0.20** | **0.69** | **1.00** | **7480** | **6102** |
| **β_z_ Period (Dispersal 2)** | **0.45** | **0.20** | **0.14** | **0.76** | **1.00** | **6801** | **6133** |
| **β_g_ Period (Dispersal 2)** | **0.47** | **0.15** | **0.24** | **0.71** | **1.00** | **6724** | **5330** |
| **β_z_ Period (Fission)** | **-0.73** | **0.20** | **-1.05** | **-0.41** | **1.00** | **5654** | **5567** |
| **β_g_ Period (Fission)** | **0.23** | **0.14** | **0.01** | **0.46** | **1.00** | **5983** | **6431** |
| **β_z_ Maternal relatedness** | **-0.40** | **0.07** | **-0.51** | **-0.28** | **1.00** | **10253** | **5726** |
| **β_g_ Maternal relatedness** | **0.24** | **0.04** | **0.18** | **0.31** | **1.00** | **8592** | **6445** |
| β_z_ Abs. rank difference | 0.01 | 0.07 | -0.11 | 0.13 | 1.00 | 9798 | 6464 |
| β_g_ Abs. rank difference | -0.06 | 0.06 | -0.15 | 0.04 | 1.00 | 7322 | 6376 |
| shape | 0.81 | 0.04 | 0.75 | 0.88 | 1.00 | 10426 | 6026 |

**Table S2. Parameter estimates from the model examining how eventual outcomes shaped female-female grooming relationships (Model 1a).** Results from a ‘hurdle gamma’ model examining the impacts of eventual outcome, sampling period, relatedness, and rank on female-female grooming relationships (n = 1521 dyad-years). β_z_ indicates parameters for the hurdle component (where positive values indicate a decreased likelihood of a zero outcome), while β_g_ indicates estimates from the gamma portion of the model (where positive values indicate an increased duration of grooming).

| **Parameter** | **Estimate** | **Std. Error** | **89% CI LL** | **89% CI UL** | **Rhat** | **Bulk ESS** | **Tail ESS** |
| --- | --- | --- | --- | --- | --- | --- | --- |
| Intercept_z_ | -1.26 | 0.41 | -1.92 | -0.63 | 1.00 | 1661 | 2861 |
| Intercept_g_ | -5.93 | 0.16 | -6.19 | -5.68 | 1.00 | 4362 | 5168 |
| **β_z_ Outcome (Split apart)** | **2.04** | **0.27** | **1.63** | **2.47** | **1.00** | **4963** | **5807** |
| **β_g_ Outcome (Split apart)** | **-0.50** | **0.15** | **-0.73** | **-0.26** | **1.00** | **8644** | **6139** |
| **β_z_ Outcome (Briefly together)** | **3.98** | **0.38** | **3.40** | **4.62** | **1.00** | **5880** | **5947** |
| **β_g_ Outcome (Briefly together)** | **-0.84** | **0.26** | **-1.25** | **-0.41** | **1.00** | **9496** | **5962** |
| **β_z_ Outcome (Never together)** | **3.89** | **0.26** | **3.50** | **4.31** | **1.00** | **8155** | **6290** |
| **β_g_ Outcome (Never together)** | **-0.99** | **0.22** | **-1.33** | **-0.63** | **1.00** | **8621** | **6442** |
| **β_z_ Period (Dispersal 1)** | **1.12** | **0.22** | **0.77** | **1.48** | **1.00** | **7479** | **6260** |
| **β_g_ Period (Dispersal 1)** | **0.44** | **0.15** | **0.20** | **0.69** | **1.00** | **7903** | **6352** |
| **β_z_ Period (Dispersal 2)** | **0.76** | **0.21** | **0.43** | **1.10** | **1.00** | **7213** | **6352** |
| **β_g_ Period (Dispersal 2)** | **0.44** | **0.15** | **0.20** | **0.67** | **1.00** | **7824** | **6399** |
| β_z_ Period (Fission) | -0.24 | 0.21 | -0.57 | 0.09 | 1.00 | 7057 | 6238 |
| β_g_ Period (Fission) | 0.15 | 0.14 | -0.07 | 0.37 | 1.00 | 7173 | 6520 |
| **β_z_ Maternal relatedness** | **-0.26** | **0.08** | **-0.39** | **-0.13** | **1.00** | **10637** | **5875** |
| **β_g_ Maternal relatedness** | **0.21** | **0.04** | **0.15** | **0.27** | **1.00** | **9860** | **6869** |
| β_z_ Abs. rank difference | 0.11 | 0.08 | -0.02 | 0.24 | 1.00 | 9697 | 6452 |
| β_g_ Abs. rank difference | -0.07 | 0.06 | -0.16 | 0.02 | 1.00 | 9099 | 6521 |
| shape | 0.83 | 0.04 | 0.76 | 0.90 | 1.00 | 10393 | 5364 |

**Table S3. Parameter estimates from the third model examining how co-residence and eventual outcome shaped female-female grooming relationships (Model 1c).** Results from a ‘hurdle gamma’ model examining the impacts of co-residence, social outcomes, sampling period, relatedness, and rank on female-female grooming relationships (n = 1521 dyad-years). β_z_ indicates parameters for the hurdle component (where positive values indicate a decreased likelihood of a zero outcome), while β_g_ indicates estimates from the gamma portion of the model (where positive values indicate an increased duration of grooming).

| **Parameter** | **Estimate** | **Std. Error** | **89% CI LL** | **89% CI UL** | **Rhat** | **Bulk ESS** | **Tail ESS** |
| --- | --- | --- | --- | --- | --- | --- | --- |
| Intercept_z_ | -1.05 | 0.48 | -1.81 | -0.27 | 1.00 | 1684 | 3481 |
| Intercept_g_ | -5.99 | 0.24 | -6.37 | -5.61 | 1.00 | 3954 | 4980 |
| β_z_ Co-residence (Same group) | -0.23 | 0.24 | -0.62 | 0.16 | 1.00 | 5129 | 6004 |
| β_g_ Co-residence (Same group) | 0.05 | 0.17 | -0.22 | 0.33 | 1.00 | 5972 | 6229 |
| **β_z_ Outcome (Split apart)** | **1.92** | **0.30** | **1.45** | **2.41** | **1.00** | **4500** | **5033** |
| **β_g_ Outcome (Split apart)** | **-0.47** | **0.17** | **-0.74** | **-0.20** | **1.00** | **7045** | **6409** |
| **β_z_ Outcome (Briefly together)** | **3.86** | **0.40** | **3.23** | **4.51** | **1.00** | **4975** | **6010** |
| **β_g_ Outcome (Briefly together)** | **-0.82** | **0.27** | **-1.24** | **-0.39** | **1.00** | **7077** | **6478** |
| **β_z_ Outcome (Never together)** | **3.68** | **0.35** | **3.14** | **4.24** | **1.00** | **4799** | **5695** |
| **β_g_ Outcome (Never together)** | **-0.94** | **0.28** | **-1.38** | **-0.49** | **1.00** | **5654** | **5936** |
| **β_z_ Period (Dispersal 1)** | **1.10** | **0.22** | **0.74** | **1.44** | **1.00** | **6446** | **6145** |
| **β_g_ Period (Dispersal 1)** | **0.44** | **0.15** | **0.19** | **0.69** | **1.00** | **7717** | **6810** |
| **β_z_ Period (Dispersal 2)** | **0.72** | **0.22** | **0.37** | **1.07** | **1.00** | **5922** | **5919** |
| **β_g_ Period (Dispersal 2)** | **0.44** | **0.15** | **0.21** | **0.67** | **1.00** | **6230** | **6146** |
| β_z_ Period (Fission) | -0.31 | 0.23 | -0.68 | 0.05 | 1.00 | 5636 | 6096 |
| β_g_ Period (Fission) | 0.16 | 0.14 | -0.06 | 0.38 | 1.00 | 5490 | 6015 |
| **β_z_ Maternal relatedness** | **-0.26** | **0.08** | **-0.39** | **-0.13** | **1.00** | **11205** | **6434** |
| **β_g_ Maternal relatedness** | **0.21** | **0.04** | **0.14** | **0.28** | **1.00** | **9853** | **5706** |
| β_z_ Abs. rank difference | 0.11 | 0.08 | -0.02 | 0.24 | 1.00 | 9473 | 6520 |
| β_g_ Abs. rank difference | -0.07 | 0.06 | -0.16 | 0.02 | 1.00 | 8791 | 6326 |
| shape | 0.83 | 0.05 | 0.76 | 0.90 | 1.00 | 11100 | 6265 |

**Table S4. Parameter estimates from the third model examining how co-residence shaped female-male grooming relationships (Model 1d).** Results from a ‘hurdle gamma’ model examining the impacts of co-residence, sampling period, male rank, and siring history on female-male grooming relationships (n = 1660 dyad-years). β_z_ indicates parameters for the hurdle component (where positive values indicate a decreased likelihood of a zero outcome), while β_g_ indicates estimates from the gamma portion of the model (where positive values indicate an increased duration of grooming).

| **Parameter** | **Estimate** | **Std. Error** | **89% CI LL** | **89% CI UL** | **Rhat** | **Bulk ESS** | **Tail ESS** |
| --- | --- | --- | --- | --- | --- | --- | --- |
| Intercept_z_ | 2.68 | 0.28 | 2.26 | 3.13 | 1.00 | 3937 | 5069 |
| Intercept_g_ | -5.87 | 0.16 | -6.12 | -5.62 | 1.00 | 6708 | 5928 |
| **β_z_ Co-residence (Same group)** | **-1.55** | **0.17** | **-1.81** | **-1.29** | **1.00** | **9638** | **6076** |
| **β_g_ Co-residence (Same group)** | **0.54** | **0.15** | **0.30** | **0.77** | **1.00** | **7453** | **6306** |
| β_z_ Period (Dispersal 1) | 0.34 | 0.27 | -0.09 | 0.78 | 1.00 | 4013 | 4734 |
| **β_g_ Period (Dispersal 1)** | **0.47** | **0.17** | **0.20** | **0.75** | **1.00** | **7739** | **6078** |
| β_z_ Period (Dispersal 2) | 0.18 | 0.30 | -0.31 | 0.65 | 1.00 | 3863 | 4302 |
| β_g_ Period (Dispersal 2) | 0.14 | 0.17 | -0.14 | 0.42 | 1.00 | 6680 | 6128 |
| **β_z_ Period (Fission)** | **-0.58** | **0.21** | **-0.91** | **-0.26** | **1.00** | **5769** | **5401** |
| **β_g_ Period (Fission)** | **0.36** | **0.15** | **0.11** | **0.61** | **1.00** | **6634** | **6513** |
| **β_z_ Male rank** | **-0.28** | **0.16** | **-0.53** | **-0.04** | **1.00** | **2989** | **4146** |
| β_g_ Male rank | 0.04 | 0.08 | -0.08 | 0.17 | 1.00 | 7589 | 5957 |
| **β_z_ Siring history (Yes)** | **-2.22** | **0.23** | **-2.59** | **-1.85** | **1.00** | **9333** | **5846** |
| **β_g_ Siring history (Yes)** | **0.41** | **0.14** | **0.19** | **0.62** | **1.00** | **8374** | **6103** |
| shape | 0.85 | 0.06 | 0.76 | 0.94 | 1.00 | 13730 | 5699 |

**Table S5. Parameter estimates from the third model examining how eventual outcome shaped female-male grooming relationships (Model 1e).** Results from a ‘hurdle gamma’ model examining the impacts of eventual outcome, sampling period, male rank, and siring history on female-male grooming relationships (n = 1660 dyad-years). β_z_ indicates parameters for the hurdle component (where positive values indicate a decreased likelihood of a zero outcome), while β_g_ indicates estimates from the gamma portion of the model (where positive values indicate an increased duration of grooming).

| **Parameter** | **Estimate** | **Std. Error** | **89% CI LL** | **89% CI UL** | **Rhat** | **Bulk ESS** | **Tail ESS** |
| --- | --- | --- | --- | --- | --- | --- | --- |
| Intercept_z_ | 0.92 | 0.26 | 0.51 | 1.34 | 1.00 | 3431 | 4626 |
| Intercept_g_ | -5.33 | 0.14 | -5.55 | -5.11 | 1.00 | 6728 | 6416 |
| **β_z_ Outcome (Split apart)** | **1.02** | **0.2** | **0.71** | **1.34** | **1.00** | **10515** | **6432** |
| β_g_ Outcome (Split apart) | -0.22 | 0.16 | -0.48 | 0.04 | 1.00 | 7717 | 5981 |
| **β_z_ Outcome (Briefly together)** | **1.18** | **0.35** | **0.63** | **1.76** | **1.00** | **9445** | **6248** |
| β_g_ Outcome (Briefly together) | -0.28 | 0.3 | -0.73 | 0.22 | 1.00 | 9611 | 5983 |
| **β_z_ Outcome (Never together)** | **2.47** | **0.27** | **2.06** | **2.91** | **1.00** | **9642** | **5435** |
| **β_g_ Outcome (Never together)** | **-0.67** | **0.27** | **-1.09** | **-0.23** | **1.00** | **5754** | **5940** |
| **β_z_ Period (Dispersal 1)** | **0.79** | **0.22** | **0.45** | **1.14** | **1.00** | **7362** | **6511** |
| **β_g_ Period (Dispersal 1)** | **0.42** | **0.18** | **0.14** | **0.7** | **1.00** | **7051** | **5595** |
| **β_z_ Period (Dispersal 2)** | **0.71** | **0.22** | **0.37** | **1.06** | **1.00** | **7092** | **6636** |
| β_g_ Period (Dispersal 2) | 0.12 | 0.18 | -0.16 | 0.4 | 1.00 | 6511 | 6253 |
| β_z_ Period (Fission) | -0.21 | 0.2 | -0.53 | 0.11 | 1.00 | 7042 | 6595 |
| **β_g_ Period (Fission)** | **0.29** | **0.16** | **0.03** | **0.54** | **1.00** | **6077** | **5979** |
| **β_z_ Male rank** | **-0.3** | **0.17** | **-0.57** | **-0.03** | **1.00** | **2952** | **4487** |
| β_g_ Male rank | 0.03 | 0.08 | -0.1 | 0.17 | 1.00 | 5930 | 5061 |
| **β_z_ Siring history (Yes)** | **-2.33** | **0.23** | **-2.71** | **-1.97** | **1.00** | **9398** | **5850** |
| **β_g_ Siring history (Yes)** | **0.48** | **0.14** | **0.25** | **0.7** | **1.00** | **10144** | **6659** |
| shape | 0.84 | 0.06 | 0.75 | 0.93 | 1.00 | 10986 | 5674 |

**Table S6. Parameter estimates from the third model examining how co-residence and eventual outcome shaped female-male grooming relationships (Model 1f).** Results from a ‘hurdle gamma’ model examining the impacts of co-residence, social outcomes, sampling period, male rank, and siring history on female-male grooming relationships (n = 1660 dyad-years). β_z_ indicates parameters for the hurdle component (where positive values indicate a decreased likelihood of a zero outcome), while β_g_ indicates estimates from the gamma portion of the model (where positive values indicate an increased duration of grooming).

| **Parameter** | **Estimate** | **Std. Error** | **89% CI LL** | **89% CI UL** | **Rhat** | **Bulk ESS** | **Tail ESS** |
| --- | --- | --- | --- | --- | --- | --- | --- |
| Intercept_z_ | 1.56 | 0.32 | 1.06 | 2.06 | 1.00 | 5048 | 5705 |
| Intercept_g_ | -5.74 | 0.20 | -6.06 | -5.41 | 1.00 | 7968 | 5905 |
| **β_z_ Co-residence (Same group)** | **-0.75** | **0.21** | **-1.09** | **-0.42** | **1.00** | **8818** | **6821** |
| **β_g_ Co-residence (Same group)** | **0.44** | **0.17** | **0.17** | **0.72** | **1.00** | **8280** | **6341** |
| **β_z_ Outcome (Split apart)** | **0.90** | **0.20** | **0.58** | **1.22** | **1.00** | **11224** | **6495** |
| β_g_ Outcome (Split apart) | -0.13 | 0.16 | -0.39 | 0.13 | 1.00 | 9039 | 5875 |
| **β_z_ Outcome (Briefly together)** | **0.90** | **0.20** | **0.58** | **1.22** | **1.00** | **11224** | **6495** |
| β_g_ Outcome (Briefly together) | -0.05 | 0.30 | -0.52 | 0.44 | 1.00 | 10550 | 6298 |
| **β_z_ Outcome (Never together)** | **1.83** | **0.32** | **1.32** | **2.33** | **1.00** | **8959** | **6748** |
| β_g_ Outcome (Never together) | -0.28 | 0.30 | -0.75 | 0.21 | 1.00 | 6847 | 5905 |
| **β_z_ Period (Dispersal 1)** | **0.79** | **0.21** | **0.46** | **1.13** | **1.00** | **8227** | **6216** |
| **β_g_ Period (Dispersal 1)** | **0.45** | **0.18** | **0.17** | **0.74** | **1.00** | **9232** | **6585** |
| **β_z_ Period (Dispersal 2)** | **0.71** | **0.21** | **0.37** | **1.05** | **1.00** | **8222** | **6035** |
| β_g_ Period (Dispersal 2) | 0.11 | 0.18 | -0.17 | 0.39 | 1.00 | 8434 | 6395 |
| β_z_ Period (Fission) | -0.32 | 0.20 | -0.64 | 0.01 | 1.00 | 7870 | 6359 |
| **β_g_ Period (Fission)** | **0.33** | **0.16** | **0.08** | **0.57** | **1.00** | **7523** | **6557** |
| **β_z_ Male rank** | **-0.37** | **0.17** | **-0.64** | **-0.10** | **1.00** | **3892** | **5232** |
| β_g_ Male rank | 0.05 | 0.08 | -0.07 | 0.18 | 1.00 | 8474 | 6175 |
| **β_z_ Siring history (Yes)** | **-2.20** | **0.23** | **-2.57** | **-1.83** | **1.00** | **11940** | **5772** |
| **β_g_ Siring history (Yes)** | **0.42** | **0.14** | **0.19** | **0.65** | **1.00** | **10878** | **6102** |
| shape | 0.85 | 0.06 | 0.76 | 0.94 | 1.00 | 14685 | 5758 |

**Table S7. Parameter estimates from the model examining whether PHG dyads that split apart were less related on average than those that stayed together (Model S1).** Results from a binomial model examining the relationship between eventual outcome (split apart vs. stayed together) and maternal kinship (i.e., maternal *r* >0, yes/no) within PHG female-female dyads (n = 136).

| **Parameter** | **Estimate** | **Std. Error** | **89% CI LL** | **89% CI UL** | **Rhat** | **Bulk ESS** | **Tail ESS** |
| --- | --- | --- | --- | --- | --- | --- | --- |
| Intercept | -1.25 | 0.43 | -1.93 | -0.61 | 1 | 5156 | 4445 |
| **β Outcome (Split apart)** | **-1.39** | **0.59** | **-2.36** | **-0.48** | **1** | **5380** | **4558** |

**Table S8. Parameter estimates from the model examining the predictors of received aggression (Model 3a).** Results from a Poisson model examining the impacts of sampling period, dispersal status, and group size on the amount of aggression females received from other adult females (n = 116 female-years).

| **Parameter** | **Estimate** | **Std. Error** | **89% CI LL** | **89% CI UL** | **Rhat** | **Bulk ESS** | **Tail ESS** |
| --- | --- | --- | --- | --- | --- | --- | --- |
| Intercept | 0.25 | 0.10 | 0.10 | 0.41 | 1.00 | 1405 | 2846 |
| **β Period (Dispersal 1)** | **0.58** | **0.05** | **0.50** | **0.65** | **1.00** | **4022** | **5516** |
| **β Period (Dispersal 2)** | **0.15** | **0.06** | **0.06** | **0.24** | **1.00** | **3705** | **5705** |
| **β Period (Fission)** | **-0.41** | **0.05** | **-0.49** | **-0.32** | **1.00** | **4404** | **5937** |
| **β Group stability (Unstable)** | **0.23** | **0.06** | **0.13** | **0.33** | **1.00** | **2876** | **4588** |
| **β Disperser? (Yes)** | **-0.26** | **0.10** | **-0.42** | **-0.09** | **1.00** | **2523** | **4176** |
| β Group size (Large) | 0.09 | 0.08 | -0.03 | 0.22 | 1.00 | 2180 | 4155 |

**Table S9. Parameter estimates from the model examining the predictors of dyadic female-female aggression (Model S3).** Results from a Poisson model examining the impacts of sampling period, social outcome, group membership, kinship, and rank on female-female dyadic aggression (n = 1521 dyad-years).

| **Parameter** | **Estimate** | **Std. Error** | **89% CI LL** | **89% CI UL** | **Rhat** | **Bulk ESS** | **Tail ESS** |
| --- | --- | --- | --- | --- | --- | --- | --- |
| Intercept | -7.28 | 0.17 | -7.55 | -7.02 | 1.00 | 1304 | 2422 |
| **β Period (Dispersal 1)** | **1.07** | **0.06** | **0.98** | **1.16** | **1.00** | **4743** | **6306** |
| **β Period (Dispersal 2)** | **0.53** | **0.07** | **0.42** | **0.65** | **1.00** | **5664** | **5893** |
| **β Period (Fission)** | **-0.39** | **0.07** | **-0.50** | **-0.28** | **1.00** | **4739** | **5236** |
| **β Outcome (Split apart)** | **-0.37** | **0.09** | **-0.50** | **-0.23** | **1.00** | **4891** | **6415** |
| **β Outcome (Briefly together)** | **-0.35** | **0.12** | **-0.54** | **-0.17** | **1.00** | **5321** | **5976** |
| **β Outcome (Never together)** | **-1.25** | **0.15** | **-1.48** | **-1.01** | **1.00** | **5356** | **5789** |
| **β Co-residence (Same group)** | **0.56** | **0.10** | **0.40** | **0.72** | **1.00** | **5388** | **5479** |
| β Maternal relatedness | -0.03 | 0.02 | -0.06 | 0.00 | 1.00 | 7802 | 6034 |
| **β Abs. rank difference** | **-0.09** | **0.02** | **-0.13** | **-0.05** | **1.00** | **7168** | **5956** |
| Zero-inflation term | 0.45 | 0.02 | 0.42 | 0.48 | 1.00 | 6405 | 5614 |

**Table S10. Parameter estimates from the model examining the predictors of the intensity of female-female aggression (Model 3b).** Results from a binomial model examining the impacts of sampling period, social outcome, relatedness, and rank on whether female-female aggression (n = 2519 events) was high intensity (yes/no).

| **Parameter** | **Estimate** | **Std. Error** | **89% CI LL** | **89% CI UL** | **Rhat** | **Bulk ESS** | **Tail ESS** |
| --- | --- | --- | --- | --- | --- | --- | --- |
| Intercept | -2.61 | 0.22 | -2.95 | -2.26 | 1.00 | 3798 | 4947 |
| **β Period (Dispersal 1)** | **0.47** | **0.18** | **0.18** | **0.76** | **1.00** | **6387** | **6326** |
| **β Period (Dispersal 2)** | **0.87** | **0.21** | **0.54** | **1.20** | **1.00** | **6133** | **6223** |
| β Period (Fission) | 0.08 | 0.21 | -0.26 | 0.43 | 1.00 | 6008 | 6069 |
| **β Outcome (Split apart)** | **0.44** | **0.24** | **0.06** | **0.82** | **1.00** | **5985** | **6415** |
| β Outcome (Briefly together) | 0.18 | 0.29 | -0.29 | 0.63 | 1.00 | 7755 | 6337 |
| β Outcome (Never together) | 0.30 | 0.27 | -0.14 | 0.71 | 1.00 | 11075 | 6001 |
| **β Maternal relatedness** | **0.23** | **0.06** | **0.13** | **0.33** | **1.00** | **9792** | **6454** |
| **β Abs. rank difference** | **0.15** | **0.07** | **0.04** | **0.27** | **1.00** | **8420** | **6486** |

**Table S11. Parameter estimates from the model examining the outcomes of female-female aggression between groups (Model 3c).** Results from a binomial model examining whether focal individuals ‘won’ a given between-group aggressive interaction as a function of her group’s relative size and the sampling period (n = 393 intergroup aggression events).

| **Parameter** | **Estimate** | **Std. Error** | **89% CI LL** | **89% CI UL** | **Rhat** | **Bulk ESS** | **Tail ESS** |
| --- | --- | --- | --- | --- | --- | --- | --- |
| Intercept | 1.06 | 0.45 | 0.35 | 1.80 | 1.00 | 3242 | 4479 |
| β Period (Dispersal 1) | -0.41 | 0.35 | -0.97 | 0.15 | 1.00 | 6780 | 5790 |
| β Period (Dispersal 2) | -0.79 | 0.58 | -1.72 | 0.13 | 1.00 | 7156 | 6536 |
| β Period (Fission) | -0.11 | 0.35 | -0.68 | 0.43 | 1.00 | 7234 | 6214 |
| **β Group size (Large)** | **1.77** | **0.27** | **1.35** | **2.21** | **1.00** | **7832** | **5813** |

**Table S12. Parameter estimates from the model examining the predictors of intergroup grooming (Model 4).** Results from a binomial model examining the proportion of individuals’ grooming budgets that were allocated towards individuals from other groups as a function of their dispersal status, the sampling period, and the sex combination of focus (n = 288).

| **Parameter** | **Estimate** | **Std. Error** | **89% CI LL** | **89% CI UL** | **Rhat** | **Bulk ESS** | **Tail ESS** |
| --- | --- | --- | --- | --- | --- | --- | --- |
| Intercept | -1.70 | 0.18 | -1.99 | -1.41 | 1.00 | 2127 | -1.70 |
| **β Period (Dispersal 1)** | **-0.75** | **0.19** | **-1.06** | **-0.45** | **1.00** | **6839** | **-0.75** |
| **β Period (Dispersal 2)** | **-0.36** | **0.21** | **-0.70** | **-0.02** | **1.00** | **5564** | **-0.36** |
| **β Period (Fission)** | **-0.26** | **0.13** | **-0.46** | **-0.06** | **1.00** | **5656** | **-0.26** |
| β Disperser? (Yes) | 0.44 | 0.36 | -0.15 | 1.01 | 1.00 | 2272 | 0.44 |
| **β Sex combo (Female-to-male)** | **0.75** | **0.14** | **0.52** | **0.97** | **1.00** | **7293** | **0.75** |
| **β Sex combo (Male-to-female)** | **-0.69** | **0.36** | **-1.27** | **-0.11** | **1.00** | **3791** | **-0.69** |
| **β Disperser x Period**  **(Dispersal 1)** | **0.75** | **0.28** | **0.31** | **1.20** | **1.00** | **6224** | **0.75** |
| **β Disperser x Period (Dispersal 2)** | **0.59** | **0.30** | **0.12** | **1.06** | **1.00** | **5878** | **0.59** |
| **β Disperser x Period (Fission)** | **1.21** | **0.20** | **0.88** | **1.54** | **1.00** | **5453** | **1.21** |
| **β Disperser x Sex combo (Female-to-male)** | **0.81** | **0.27** | **0.38** | **1.23** | **1.00** | **7323** | **0.81** |
| **β Disperser x Sex combo**  **(Male-to-female)** | **0.91** | **0.55** | **0.05** | **1.79** | **1.00** | **2724** | **0.91** |
