# Supplementary material for: Social patterns underlying a new group formation in olive baboons
Source: PLoS One. 2025 Oct 22;20(10):e0333541. doi: 10.1371/journal.pone.0333541 (PMC12543123; doi:10.1371/journal.pone.0333541)
Supplement: Attachment — Submitted filename: R1 Response to Reviewers.docx [file pone.0333541.s003.docx]

**Editor Comments**

This study analyses affiliation and aggression networks prior, during and after a single group fission event in olive baboons. While previous studies mainly looked at patterns of group fissions, this case study of a male-initiated fission is really interesting, as it provides detailed analyses in the process of group split/s. Hence the study is providing an important foundation for comparative work of group fissions in nonhuman primates. Even though the process seems to be quite unique (male-initiated) from what has been described previously, the results compare well with previous studies, i.e. that females stay with their closest social partners which are close maternal relatives.

The manuscript has been reviewed by three experts and myself. The manuscript is well written and I enjoyed reading it. However, I asked a third reviewer to explicitly evaluate the statistical approach (given the complexity of the analysis). All reviewers raised several important concerns and I agree with them in all points. While the topic is really interesting and the data are sufficient to test the questions raised, the current version of the Ms. has serval serious limitations which I summarize below:

1. Introduction: would benefit from details on fission types observed in nonhuman primates (apart from male- and female-initiated splits). I think the main pattern are that splits occur across and between matrilines, while mainly females remain with close maternal relatives.
2. Methods: most reviewer comments are related to the statistical models as they are hard to follow and often less clear. You run a total of eight Bayesian models and five frequentist analyses and it is unclear why you think they are necessary to address your questions. You nicely provide several questions, however, I recommend to link each question with one or more model/s. Furthermore, each analysis should be introduced separated while ideally being numbered (and/or list a short title), so the readers can view the methods and results per model in more detail. You can also think about moving some analytical parts of lower importance into the supplement to increase readability of the main Ms. In the current stage, the analysis seems to have several issues which need to be addressed in order to present it in a way readers can potentially digest it.
3. Results: the results are difficult to follow. While they are linked to the broader question, they are not link to individual model/s. As pointed out by reviewers, please provide tables with model outcomes for all predictors and random effects including information on sample size and distribution of predictors.
4. Conclusion: It is recommended to restrict conclusions to the actual results you are presenting.

In the light of these points, a major revision of the Manuscript is needed. If you are willing to provide a complete revision of the Ms, I will be happy to revise my decision and suggest the publication of this Ms. in Plos One. I think the study will be a valuable contribution to the literature.

>> Thank you for the opportunity to revise our manuscript. The manuscript, its results, and its presentation are much improved in light of reviewers’ comments.

Regarding the Introduction, we have revised our description of the two main ‘types’ of fissions. We agree that fissions that preserve vs. break maternal kin relationships are a meaningful distinction, which we now highlight more throughout the Introduction. In general, our intention was to group fissions by their probable proximate triggers (i.e., feeding competition vs. male mating competition) and not by their demographic outcomes. We hope our rewording emphasizes this intended focus.

In the Methods section, we now delineate our models more clearly, using headers that match the ordering of our research questions, as delineated in the Introduction. We have also removed many of the frequentist analyses, provided descriptive data where these would suffice, or moved to Bayesian approaches where we felt these were appropriate (L318-323).

In the Results section, we carry this organizational scheme forward. We also provide complete model output tables in the Supplementary Materials.

Lastly, we have removed the Conclusions section, as it was largely redundant with material provided elsewhere.

**Reviewer #1:** In this manuscript the authors describe changes in social behavior around a single group-fission event in a population of olive baboons. As a stand-alone case study the analyses will gain value only when others provide similar data fueling future statistical evaluation of the phenomenon. This being said, the manuscript provides a detailed account of the affiliation and aggression network before, during and after the fission event which is rare but desirable. The manuscript would benefit from a thorough revision of the intro where the two types of fission events observed in primates are defined and in the sequence of presentation of material. There is a glitch in the statistical analyses that needs fixing and discussion would benefit from relating the findings to a recent analyses of yellow baboon group fission events relative to female social bond networks. Below I provide comments as they came up during my first reading of the manuscript which mimics the experience of a future reader.

Introduction

Line 42: It may be noteworthy that in marginal habitats chacma baboons have been reported to form one-male groups and that some colleagues believe that these are the conditions during the speciation of chacma baboons that have left a trace in current day social behavior patterns and reproductive strategies including infanticide. As such, chacma baboons would fall within the same group as gorillas and Asian colobines – they have it in them to form one-male groups.

>> We agree that these chacma baboon fission products are quite relevant here. We now elaborate on this point in L46-48.

Line 51: The example does not really illustrate the point that individuals have different goals, because the male priority to recruit a number of females does not have to be at odds with females maintaining their close affiliative relationships if an entire matriline joins the event.

>> We’ve reworded this example to better illustrate this point in L54-56.

Line 56: The reader would benefit from a more detailed description of the published data on group splits in baboons and particularly those that are classified as male initiated. It is not entirely clear which criteria are used to classify events into male vs. female led. Is it the temporal pattern, a male goes first and the females join later? Is it a male actively herding females away from the main group? Is it always only one male or sometimes more than one? Is it not true that any number of females that start a new group will always do so together with one or several males?

>> We’ve revised this passage for clarity. In short, we used this schema to highlight two ‘triggers’ that instigate fission in primates (i.e., female-female feeding competition, and male-male mating competition). while male-driven fissions are those that maximize the instigating male’s reproductive opportunities. However, we note that these categories are not mutually exclusive and inferred by both circumstantial (i.e., the temporal patterning of individual social decisions, the occurrence of male ‘herding’ behaviors) and quantitative lines of evidence (i.e., whether females were able to preserve their kinship ties).

Applied to the current study: What tells us that it was Yohan who led the females and not the females that led Yohan to ENK? Three females and Yohan went to ENK, 1 female and Yohan stayed. The 2 females that went back, retrieved another three to join ENK for a second stint and then decided to do their own thing for which they were joined by Yohan. The story could be told from the perspective of those two females that checked out opportunities, then mustered supporters to join ENK which failed and motivated these females to start a new group.

>> We now provide a more detailed timeline at the beginning of the Results section. Specifically, Yohan first moved into ENK along with three females on January 2015. Thus, at this temporal scale, we cannot say which preceded the other. We now note this in L297-304.

Methods

Kinship: It is not clear whether kinship was modeled as a continuous, dichotomous or a multinomial variable one and how this may have affected the results. On line 291 it seems that relatedness was classified into close kin versus non-kin, i.e. two categories? The data on github suggest a more fine-grained measure.

>> We now clarify this fine-grained measure and provide a more succinct description in L137-140.

It seems that the statistical models do not include random slopes (e.g. the effect of group may vary across subjects or the effect of period may vary across subjects) and their correlations with random intercepts which can drastically increase type 1 error rate. Barr et al 2013 Random eﬀects structure for conﬁrmatory hypothesis testing: Keep it maximal. J Memory and Language, 68:255–278; Schielzeth& Forstmeier 2009 Conclusions beyond support: overconﬁdent estimates in mixed models. Behav Ecol 20:416–420.

>> We agree in principle that including random slopes would allow us to determine whether the effects of co-residence and eventual outcome (i.e., our two key predictors) vary across study subjects. However, given the broader complexities of our modeling approach (i.e., multi-membership random intercepts, hurdle gamma models which estimate two parameters for each predictor), random slopes did not converge for our primary models focused on dyadic grooming (i.e., Models 1a-f). We now state this in L195.

Likewise, the key metrics of interest in our other models (i.e., dispersal status, yes/no) did not vary within individuals. Thus, random slopes would not be informative in these cases.

Results

During my first reading I was not entirely sure about how females were assigned to a group. In Fig. 3 the estimated grooming time per hour for individuals that never stayed together clearly differs from zero and is just about ¼ of what you get if individuals lived in one group throughout. Where do you draw the line if there is so much grooming between members of different groups? This is even more extreme for between-sex grooming. Is that typical for the population, the species, baboons in general to see member of different groups grooming regularly? Why are all posterior estimates in the text negative?

>> Group membership was determined during routine observation censuses, and individuals were assigned a monthly group membership based on the majority of these assessments. We state this outright in L95-97.

This is certainly atypical for baboon groups but likely stems from a recent history of fission. We reiterate this point in L68 and L485-489.

As a reader I would prefer to see a table with model outcomes for all predictors and random effects including information on sample size and distribution of predictors (e.g. number of close kin and non-kin dyads).

>> We now provide a thorough set of model output tables in our Supplementary Materials.

Line 295: Something is missing here to indicate which estimate is for which of the groups compared. From the text is looks like dyads that stayed and those that separated were compared with some Wilcoxon Test. Since these dyadic data include repeated measurements the data points are not independent and we would look at a case of pseudo-replication inflating Type I error.

>> This is a fair concern. The initial analysis was meant to serve as a *post hoc* check on whether Yohan’s relationships predicted these choices in a more targeted fashion. To alleviate these concerns, we’ve removed many of these *post hoc* analyses, included simple descriptions of these data where they remain useful, or switched to Bayesian approaches that capture data interdependence where we feel this approach would be both productive and applicable.

Line 296: This needs more explanation to make the analysis more accessible in the results section. How can there be as much grooming between females that currently live in the same and in different groups? Why are females grooming outside their group so much? Who is compared to whom here? The issue applies to analyses of female-male grooming and of aggression. Between-group grooming is mentioned in the methods section but appeared to be 10 times less frequent than within (600 vs. 5400 events).

>> Apologies for the confusion, as our original visualization overstated the extent of between-group grooming due to our confounded variables. We also introduce the prevalence of between-group grooming earlier in the Introduction (L68-70) and provide potential explanations in L486-490.

Line 357: there is a word missing after slightly to indicate whether contact aggression was more or less likely.

>> This has been corrected.

Line 365: Perhaps you want to move this part about grooming between groups up to the beginning of the results section, because as you see from my comments, this amount of BG-grooming is unusual and very confusing to read about. The discussion talks about a priori knowledge on BG-grooming since 2011 which is info that could be moved all the way to the introduction.

>> Same as above.

Line 400: It is unclear how this test was run and how data were pseudo-replicated here as indicated by the very large W-value. This would become immediately clear if the sample sizes were reported with the test statistic and p-value.

>> Due to other reviewer concerns, we have removed this analysis and opted to simply state that many of the dispersing females belonged to a single matriline.

Line 409: The conclusion that females were influenced by Yohan and their female kin seems a bit overstated. What is the evidence that rules out that females made their own decisions and Yohan and (and a bunch of subadult males) just joined the move? If as stated here, Yohan was natal to PNG, his natal dispersal had to come at some point anyways and thus may not have been driven by male-male competition but by incest avoidance.

>> We now state outright that inbreeding avoidance is the likely explanation for why Yohan left his natal group, PHG (L459-460).

The conclusion paragraph is redundant with the introduction and can be cut from the manuscript.

>> Given these redundancies, we have removed this paragraph.

It would be great to discuss the results in relation to the Better baboon break-ups analyses by Lerch et al (a study cited here).

>> This is a useful study to reference here. We now elaborate on these data in comparison to ours in the Discussion (L464-466).

**Reviewer #2**

This was a detailed look at a group fission event, which provided insight into possible factors that influenced decision-making with regards of staying in a group, or leaving it. It is a nice manuscript to read, and I certainly feel I learned a lot about fissions and factors influencing who stays together.

However, I feel the paper would have benefited from more anecdotal information about the event, as this is primarily a case study of a fission. For example, what is missing is also more descriptive information about the initial dispersal of Yuhon. Once he dispersed, did the females immediately follow? Was there a brief period when the females stayed in their group (PHG) before following Yuhon? The female that stayed with Yuhon throughout Dispersal 1 and Dispersal 2, was there something different about her relationship to Yuhon or her female kin in PHG? What was different in the circumstances of the 1 female that did not remain with Yuhon?

>> We now provide a more detailed timeline at the beginning of the Results section (L297-304). Unfortunately, due to the coarse timescale at which group membership is determined in this study population, we cannot say whether there was a ‘lag’ between Yohan’s dispersal and the three females’ dispersal. We also note that the female who remained in ENK throughout the remainder of the study had kin within this group, which may have contributed to her social decisions (L300-302).

The main take-home in the end seems to be that prior mating/siring history is what influenced the decisions of females to stick with Yuhon, and that these females were also closely related to each other. These are not captured strongly in all the many models in the paper, which makes me also wonder if the models were unnecessarily filled with too many fixed effects. Without any mention of how the different fixed effects influence each other (no DAGs), its less clear how to interpret each fixed effect estimate from each model. For example, maternal relatedness should definitely have a causal role in determining eventual outcome.

>> Thank you for this valuable point. We have made two adjustments in response. First, we now provide DAGs as visualizations of our predictions in the supplementary materials, indicating the expected relationships between our fixed effects. This DAG indicates that adjusting for rank and maternal kinship closes ‘backdoor paths’ that precede social decisions and their impact on grooming behavior. Second, we now include recent paternities as a covariate in our model examining female-male relationships, as this is what we expect to be driving female-male grooming and grouping decisions. Thank you for raising this important point.

I feel this paper would have been easier to interpret had a model been focused on using data from the Stable period to predict the eventual outcome.

>> We wholeheartedly agree. Our analytical choices were largely a byproduct of the fission’s complexities, such as the piecewise dispersal of females from their natal groups into a neighboring group and, eventually, a new group. Our goal was to document these idiosyncrasies and quantify the fission as a process and not a singular event. By default, this decision sacrificed interpretability in order to create a more holistic picture, which we believe remains useful. Nevertheless, we have edited to explain this approach and clarify our results.

I didn’t find any reference to the total number of focal subjects or the total number of dyads for the study. These numbers should be stated early in the Methods section (perhaps even the abstract), instead of the reader having to run code or look through SI information to find out.

>> Our apologies. These are now listed explicitly in the Methods.

I did not have access to the SI information, but please state in the main manuscript how model fit was assessed. Your SI material should include poster predictive checks, and trace plots to show good mixing of chains. What were considered acceptable rhat values and minimum effective sample sizes?

>> We now list more details regarding our models and how these were assessed in L290-294.

More specific comments:

Lines 84-87: Here would be the best place to introduce the information that intergroup- grooming continued to be common even after fissions. This did not come up until the discussion. This led to my confusion later (methods) as to why you would analyze data from dyads not co-resident in the same group.

>> Thanks for this suggestion. We now mention the prevalence between-group grooming in the Introduction (L68-70).

Lines 120-122: “An additional four females dispersed from PHG to ENK at the start of the Dispersal 2 period”. I am trying to reconcile this with the information in lines 99-100 which states that “two females that had returned to PHG moved back into ENK along with three other females”. Isn’t this then “An additional three females”?

>> Thank you for spotting this error. We’ve corrected these numbers of consistency in L128-129.

Line 125: the sub header “kinship” should be changed to “maternal relatedness” as the manuscript does not touch upon kinship relationships, only expected relatedness levels along the maternal line.

>> Thank you for this suggestion. We’ve now changed this term to maternal relatedness throughout.

Lines 136: section on dominance. It is not clear why dominance has come up. It is not mentioned as a focus point in the introduction and suddenly appears here. Are there predictions for the influence of dominance rank? Or as it turns out rank difference in the models? The authors have not set up why it is important to consider in this study.

>> This is a fair point. We now clarify why we chose this measure in L215-219.

Line 151: Please include here the period over which these >14000 observations were taken. Yes, its in Figure 1, but it should also be mentioned here.

>> We now provide the dates of the study period in L162.

Somewhere in the section also please state the number of unique dyads in your dataset, broken down also by female-female, and female-male subsets.

>> We now provide this information in L214-216.

Line 152: Please indicate 1) number of females that were focal-followed, and also the 2) number of males that were focal-followed..

>> We now provide this information in L162-163.

Lines 166-180: If I am understanding this correctly, each ‘edge weight’ here is a point estimate. All the information about variation in sampling density is lost at this stage. The rest of this manuscript mentions Bayesian statistical approaches, so why not use Bayesian approaches here? With “nine networks for each behavioral type” (n=18 networks?), how much of the variance between networks in this manuscript is going to be due to noise because of differences in sampling effort. Figure 2 has no uncertainty around its point estimates.

>> This is a very fair concern. To incorporate uncertainty into our presentation of these broader network-level changes, we use the results from the dyadic regression models to generate posterior networks and calculate these metrics. This required us to rearrange the order of these analyses, as the creation of these networks hinges on our dyadic model outputs. We describe this process in greater detail in L231-243.

Line 183-185: The authors state that they use a hurdle gamma model. 1) Please state explicitly that you do not use separate formulae for the hurdle and the gamma part. 2) You should give a brief description of what a hurdle gamma model is. Yhe average reader is not likely to know.

>> We now provide a short description and more clarification in L180-190.

Lines 201-202, 222, 237-238: the authors will need to be explicit about what they mean by including “multimembership random effects term for dyad”. The average reader will have no idea what you mean by this. The model will return random intercept terms for Subjects, correct?

>> That is correct, and our original wording was misleading. We’ve reworded this in L193-195.

Statistical analyses:

This section was tough to get through on the first read through, given the various covariates and how they differed depending on the specific model. Then linking later results to specific models was time consuming. Please name each model in the statistics section, and bring back that model name in the dissemination of results.

>> Thank you for this suggestion. We now provide numbers for our primary models and link these to our specific research questions and through our headers throughout the Results section. We hope this improves the legibility of these analytical approaches. We have also pared down our redundant analyses in order to make this more digestible for readers.

State explicitly when you are using undirected data in your analyses. In most cases, you have aggregated by unique dyad (removing directionality of behaviour in most analyses). Though your data is directional in its raw form, it is no longer directional in most of your aggregated data tables that are fed into your models.

>> We now mention that we’re using undirected data for these grooming analyses in L179.

Please indicate what your reference categories are for analyses to make interpretation of results easier:

Outcome (4): stayedTogether, splitApart, BrieflyTogether, NeverTogether

Co-residence (2): Same/Different group

Sampling period (4): stable, dispersal1, dispersal2, fission

>> We now list our reference categories in L311, L376, 393, 404, etc.

I struggled a bit when considering the relationship between co-residence and outcome. Am I correct in saying that dyads in the StayedTogether category are also always co-resident (Same category), and dyads in the NeverTogether category are also always not co-resident (Different category). So, co-residence only has variation in the splitApart and BrieflyTogether categories. So, really the interesting comparison would only be: do splitApart and BrieflyTogether dyads act differently when they are co-resident versus not (interaction).

>> That is (mostly) correct. By and large, the dyads within the StayedTogether category were co-resident throughout the study, except for the handful of females who dispersed earlier into ENK but rejoined their original groupmates later on. However, you are correct that the NeverTogether dyads never overlapped at all. We agree that this more targeted comparison would be useful.

To alleviate these concerns regarding these correlated predictors, we’ve now changed our modeling approach, running separate models examining the influence of (i) co-residence, (ii) eventual outcome, and (iii) co-residence and eventual outcome. We then use model comparison approaches to determine which model provides the best fits for female-female and female-male grooming relationships respectively.

Lines 215-216: this wording makes it unclear to me whether by “dyad-level” you mean 1) the total amount of aggression the dyad received in aggregate (sum of aggression to id1 and id2) or, 2) the total amount of aggression directed within the dyad (at each other, by each other).

>> Good catch! Given feedback from the Associate Editor, we have now moved the dyad-level analysis to the Supplementary Materials and have reworded this to better reflect our analysis.

Line 220: what is “current group combination”?

>> We simply meant co-residence. We’ve reworded this for clarity.

It is unclear to me why the individual-level version of the aggression model is necessary, as this is the same aggression data that is better represented in the dataset of the dyad-level dataset (an individual might at one extreme receive a lot of aggression from one individual, that drives their estimate up even though they receive little aggression overall from other baboons).

>> That is a fair point. Our main goal with the individual-level analysis was to determine how aggression changed in the aggregate. Given that this is our primary focus, we now present the individual-level models alone in the Main Text. However, we still include the dyadic-level models in the Supplementary Materials.

Figure 1b: I always love a good Sankey plot for this sort of information. As a service to the reader, perhaps mention what this type of graph is called, so that the reader can look up information on its use for visual representation of change in composition (in case they want to make one themselves in the future). It’s a very nice figure.

>> This is a good suggestion. We now include this information in the Figure Legend (L126).

Results:

Lines 259-270: This section is hard to interpret because there is no indication of the strength of the evidence for these statements. As mentioned before, these appear to be based on point samples that discard uncertainty in their estimation due to variation in sampling density.

>> As mentioned above, we now have modified these analyses to account for these methodological uncertainties.

Lines 282-288: Unsure which analysis these results come from. The info is under “grooming networks” but sounds like the results are from the grooming hurdle gamma model. Where do these estimates come from? I think these are the estimates for Period in the grooming hurdle model. But I am confused because here it mentions that “we did not include these interaction terms in the models described below”. So, but these results do include an interaction? (so a separate model?). Please clarify.

>> Our apologies for the confusion. This placement was mainly to reiterate what we mentioned earlier in the results (i.e., that interaction effects did not improve model fit). We’ve now removed this text for clarity.

Line 423: include here what counts as a close female relative (matR >= ?).

>> Thanks for catching this omission. We used matR ≥0.0625 as our criteria.

**Reviewer #3:** This manuscript describes and quantifies the demographic and behavioural dynamics during a group fusion and subsequent fission event in a population of olive baboons. The authors present an impressive data set, including affiliative and aggressive interactions of all adult animals in three social groups, collected over the course of several years. The group fission was initiated by a single adult high-ranking male, who first migrated to another group and later formed its own new group. Several females followed him. The authors found that those females had close social bonds with this male prior to his dispersal. Furthermore, these females had offspring sired by the dispersing male. The authors further found that the dispersing females were not evicted from the group, suggesting that dispersal was their active decision. Additionally, females tended to remain in the same social group as their closest grooming partners.

While the research questions are interesting and the data set to answer those is very comprehensive, I have a few concerns that should be addressed before publication. Please note that I was asked by the editor to specifically review the statistical analyses, rather than the whole manuscript.

Generally, I found it quite hard to follow the description of statistical analyses that have been conducted. To my understanding, the authors report a total of eight Bayesian models and five frequentist analyses. Especially in the results section I was struggling to understand from which model the reported numbers originated. That is why I would strongly suggest numbering the models consistently throughout the Methods and Results section, as was started in L214-215. Furthermore, the frequentist analyses came as a surprise to me in the Results and Discussion, as they have not been mentioned anywhere in the Methods. Please explain why and how you ran these tests in the appropriate sections.

>> In response to another reviewer’s suggestions and to make it easier for readers to follow our analyses, we now provide headers for each primary question listed in the Methods, number these models accordingly, and repeat these labels throughout the Results section.

We also appreciate the concerns regarding our frequentist analyses. We intended for these to largely act as “back-of-the-envelope” calculations to provide estimates that are often provided in descriptions of primate fission (i.e., increases in maternal relatedness, etc.). To alleviate these concerns, we have modified this approach by (1) removing analyses where descriptive data would suffice, and (2) switching to Bayesian methods where we think these are appropriate and helpful.

L137-140: It is not clear to me if the ranks were calculated each month separately or if the ranks of the previous month were used as input to calculate the rank of the next month. Please clarify.

>> We’ve reworded the text for clarity. We simply meant ranks were calculated monthly based on (1) each individual’s Elo-rank in that month and (2) the resulting proportion of individuals that they outranked in that given month.

L143-144: Did female ranks remain stable, even if they changed group membership?

>> We’ve reworded the text to clarify that, while relative ranks were stable, proportional ranks did change in response to demographic change (L154-156).

L151-154: Please mention how many females and males the data set includes in total.

Bayesian models: What is the reason you did not include random slopes in your models?

>> We now provide sample sizes in L163 and L214-215. We agree in principle that including random slopes would allow us to determine whether the effects of co-residence and eventual outcome (i.e., our two key predictors) vary across study subjects. However, given the broader complexities of our modeling approach (i.e., multi-membership random intercepts, hurdle gamma models which estimate two parameters for each predictor), random slopes did not converge for our primary models focused on dyadic grooming (i.e., Models 1a-f). We now state this in L195.

Likewise, the key metrics of interest in our other models (i.e., dispersal status, yes/no) did not vary within individuals. Thus, random slopes would not be informative in these cases.

L185: Could you please indicate what percentage of the response values were zero?

>> We now provide this information in L180-182.

L197-198: Would it not be interesting to see if kin biased social preferences play a role as well in the grooming dynamics over a fission event?

>> Given that olive baboon males regularly disperse from their natal groups, very few dyads showed maternal relatedness >0. Given this paucity of information and our focus on mating relationships in guiding the fission process, we removed these dyads.

L200-201: How was the total dyadic sampling effort quantified?

>> Total dyadic sampling effort reflected the sum of focal observation hours for both individuals within the dyad. We now state this outright in L191-192.

L203-210: Curiosity question: Did the authors try to improve the model fit by increasing the iterations, delta and the maximum tree depth? It seems that the default settings of brms worked for all other models (L254-256). However, adapting the parameters could solve the convergence problems and enable them to answer these very interesting questions.

>> We now clarify that our reasoning for not including the interaction effect models was not based on convergence but through leave-one-out cross-validation, which indicates that including interaction effects does not improve model performance. Thus, we report the simpler main effects model in the main text.

L213-238: The authors describe four models to test for aggression dynamics in females. Only one out of four models accounts for sampling effort and I wonder why this was not done for all four?

>> This is because the other models were analyzed on an event-by-event basis. In other words, given an aggressive event occurs, what factors influence (Model 3b) whether it escalates to physical contact or (Model 3c) whether the focal individual ‘loses’ during a between-group interaction. We reiterate this in L263-264.

L218-219: Why was the group size included as a categorical measure rather than the actual size?

>> We did this because group size was rather discontinuous and bimodal across the sampled groups.

L220: What is “current group combination”? Does this measure indicate if the individuals are in the same or a different group? Or did the authors specifically include in which groups the individuals were?

>> We simply meant current co-residence (yes/no). We’ve reworded this for clarity.

L228-231: I wonder why the authors did not include the current group of residence as a fixed effect in the model, as the groups might differ in aggression dynamics. Please clarify.

>> We did not include the group of residence as a fixed effect, as this varied for both individuals within the dyad.

L240-244: Why did the authors chose a zero-inflation rather than a hurdle model in this case? Also, it is not clear to me if the reported error distribution was applied to both models or just the latter. Please clarify and report what percentage of the response values were zero.

>> We opted for a zero-inflated model because the data were Poisson-distributed counts and not Gamma-distributed durations. Unlike duration data, these count data more readily lend themselves to zero-inflation models in ‘brms.’

L282-319: The authors report that they performed hurdle models for the grooming data, as it contained an excess of zeroes (L183-185). A hurdle model consists of two parts: A hurdle and a count part. The hurdle part treats the response as binary variable and estimates which predictors influence that the response is unequal to zero. The count part of the model only takes observations into account where the response is unequal to zero and estimates which predictors influence the magnitude of the response. However, in the results section, the authors only report one estimate per predictor and do not specify if that originates from the hurdle or count part of the model, which would be essential for interpretation. Please clarify.

>> We clarify that these estimates in the Main Text pertain to the second component of the hurdle model (i.e., where values >0). However, we do this solely for brevity and state that these directional estimates are concordant with the ‘hurdle’ portion of the model. We also provide parameter estimates for both portions of the model in the Supplementary Materials.

L295: Please specify what the numbers in brackets refer to.

>> Our apologies for this error. The original mathematical symbols had disappeared due to a conversion issue. In light of feedback from other reviewers, we have also removed these non-parametric statistics.

L318: How can β be equal to one edge of the CI?

>> We’ve removed this from our analyses and thus this typo has been removed.

L325-328: I wonder if this question could have been answered by including related dyads in the female-male grooming model (L183-198) instead of running an extra test?

>> See our note above regarding female-male related dyads.

L368: Should it not say βdisperser x stable?

>> We now clarify that the Stable period was our reference category here in L404-405. Thus, this parameter estimate βdisperser pertains to the effect of dispersal status during this reference Stable period.
