# Supplementary material for: Social patterns underlying a new group formation in olive baboons
Source: PLoS One. 2025 Oct 22;20(10):e0333541. doi: 10.1371/journal.pone.0333541 (PMC12543123; doi:10.1371/journal.pone.0333541)
Supplement: Attachment — Submitted filename: R2 Response to Reviewers.docx [file pone.0333541.s004.docx]

**Editor Comments**

The revision was seen by all three previous reviewers. They all agree that the Manuscript improved substantially. Two reviewers still have some smaller clarification issues or ask for visualization of the posterior distributions of model estimates (which I agree would be very helpful to understand your results better) and my comments have also been incorporated well. However, the third reviewer still has an analytical issue. The question is: do you go with those you got along well in the past OR do you prepare for a group fission by deepening certain relationships? These might be two different things assuming that monkeys could anticipate an upcoming fission event. It may also be difficult to analyze all these phases of this complex process in one model, because these females may have different problems to solve in each phase, e.g. who to join when a fission occurs or who to groom in a new group for successful integration. I think the reviewer may have a point here, which was not really clear in the original version. Therefore, I need to ask you to address these concerns before I can suggest this Ms. for publication. I decided to give you another major revision as this will provide you with more time, however, you can of course submit earlier.

>> Thank you for these comments, which have pointed us towards a more thorough explanation of our objectives in the introduction. Our aim in modeling social relationships across these four time periods was not to see whether individuals that stayed together deepened their relationships *per se* but rather to see (i) whether individuals that split apart weakened their relationships in the period prior to their departure and (ii) whether the dispersing individuals from PHG strengthened their intergroup grooming relationships with ENK residents in anticipation of their dispersal (i.e., whether they engaged in ‘shopping’ before their dispersal). Although we had considered modeling this sequence via two separate models (i.e., first the females’ initial dispersal using dyads from PHG, second the dispersing females’ failure to integrate using dyads from ENK), we ultimately opted for this complex model in order to detect these subtle patterns. We’ve revised the text in the Introduction (L70-73) and Methods (L200-206) to make this reasoning and framework clearer, adding references to previous research suggesting that primates prune their social ties in the years leading up to fission.

That being said, the lack of support for interaction effects makes it clear that this was not the case. In other words, individuals did not shift their social preferences as they faced different social problems across each sampling period, and individuals did not subtly begin to groom beyond their social group in anticipation of their dispersal. Instead, individuals simply stayed with those they got along with in the past. We now comment on this result and its implications more explicitly in the Discussion in L465-468.

Two smaller comments:

L 195: you used no random slopes at all? Usually you still include all identifiable random slopes if your random correlations cause convergence issues. Pls specify.

>> We now clarify that we did not use random slopes for our primary factors of interest (e.g., sampling period, eventual outcome) because there were not multiple observations for each category for each individual (L191-193). For instance, some females were not present during specific periods (because they disappeared or matured midway through the study) and not all eventual outcome types were available for each female (by definition, females that never dispersed did not belong to any *briefly together* dyads), and thus we were unable to reliably estimate random slopes for these categorical variables.

L 219: related m-f dyads, you mean mother-son or father-daughter dyads and maternal siblings? Pls specify.

>> This is a fair point for clarification, as we only know maternal kin from demographic records, and genetic sampling is relatively shallow (i.e., we may have inadvertently included unconfirmed father-daughter dyads). We’ve modified the text to clarify that we only removed maternal kin and now provide sample sizes for the omitted maternal kin dyads in L235-236.

**Reviewer #1:** I came away from reading the revised version with a better understanding of the analytical strategy and new concerns about its appropriateness. Firstly, the DAGs provided in the supplement scrutinize the causal hypotheses underlying the statistical models. I do not share the view that grooming relationships are a causal consequence of fission outcome. There is no theoretical reason or empirical work suggesting that females form closer relationships with particular females in anticipation of an upcoming fission event. These grooming relationships form whether the group fissions or not. And when it is time to decide which daughter group to move to, females go with their close partners or the majority of their close partners, i.e. the causality is reversed and grooming drives fission outcome. The causal relationship between co-residence and grooming relationship is straightforward: if we do not live together, we do not see each other and cannot groom. In the study population groups seem to aggregate enough to provide some, but surely not equal grooming opportunity for those living in the same and different groups. The causality between co-residence and fission outcome is less clear.

>> Thank you for this comment. We agree that grooming relationships are not a causal outcome of fission and the DAG presented in the supplementary materials obfuscated this presumed relationship (i.e., that demographic outcomes following fission are the consequence of grooming relationships). Our intention in using fission outcome as a predictor was to capture the individuals’ latent social preferences (which are unmeasurable). In other words, current group membership is a reflection of the females’ past social preferences, while eventual outcome is a reflection of the females’ ongoing social preferences. This eventual outcome categorization may account for variation above and beyond that associated with current group membership. As far as how co-residence is linked to fission outcome, we merely meant to indicate that individuals who are co-resident are more likely to end up in the same groups than individuals who are not co-resident.

We now comment on these causal relationships more explicitly in Figure S1 and its caption and provide more reasoning for this approach throughout the Main text.

Secondly, the way data are analyzed across groups may be confounded by very strong co-residence effects that blur the interesting facts. If the 2-3 groups are treated as one large network as suggested by the network graphs, all analyses will reproduce who is living in which group in each study period. Every dyad was labeled as one of the four fission outcome classes and their grooming relationship measured four times, once per study period. Dyads that always stay together necessarily have a stronger grooming relationship across the four periods than other dyads, because members of the other dyads did not live in the same group in at least some of the periods (666 out of 806 values for dyads from different groups are zero compared to 317 out of 715 from the same group). If a dyad lived together only briefly, then this brief period is when their grooming should have been measured. The way the data are analyzed does not tell us how the dispersing females did or did not integrate into the grooming network of ENK during periods 2 and 3, or why one female stayed in ENK while other dispersers did not. In the current analyses, this information is now hidden behind very strong patterns driven by co-residence.

>> We agree that modeling these two correlated variables creates problematic complexities due to confounding. These concerns motivated our choice during the previous revision to construct and compare models including (i) co-residence alone; (ii) eventual outcome alone; and (iii) co-residence and eventual outcome combined to determine whether these two variables capture non-redundant information within the dataset. While modeling co-resident dyads alone would be a more focused representation of the fission process, we chose the current approach because we wanted to know whether females engaged in ‘shopping’ prior to their dispersals (i.e., whether they increased the amount of grooming of outgroup individuals before making the decision to leave). To capture these potential temporal patterns, we included interaction terms between study period and the eventual outcome categories. However, the interaction term did not improve model fit (in models that both did and did not include co-residence as an additional term), which suggests that these temporal changes did not occur. In other words, dispersing females did not integrate into the broader group, as the “briefly together” dyads formed weak relationships across all of the sampling periods (even during their periods of overlap). Likewise, dyads that split apart did not weaken their relationships in anticipation of their split.

I list my other comments as they came up during my first reading of the revised ms.
Please note that the line numbers I use here refer to the manuscript version with tracked changes.

Line 12: Please consider rephrasing. It does not seem to be adequate to say here that females followed subsequently if the male went at the same time as the females. On line 123 and in the response letter it sounds like the male went TOGETHER with three females and on line 381 of the results section it is clearly stated that it is unknown whether the male went first.

>> Thank you for this suggestion. We’ve modified the text in L11-12 to avoid this false impression.

Line 15: Together with the next sentence this is difficult to parse for a first time reader. Is the sentence only about females that remain, i.e. do not disperse, or also about the females that did disperse and did so with their main female grooming partners. The same ambiguity applies to the co-residents in the next sentence. Are co-residents the individuals that did not disperse or those that end up in the same group at any step throughout the dispersal event? In the abstract it may be useful to use one word for one thing to better control what the reader takes home.

>> We’ve modified the text to be more consistent and more closely match our modeling approach.

Line 45: There is no first to this “Second, …”

>> Thanks for pointing this out. We’ve removed this.

Line 50: The references do not seem to support the statement here. Langurs in Jodhpur (Winkler et al.) live in one-male groups and are subject to take-over from bachelor groups and the Thomas langurs Sterck studied disintegrate when the single resident male cannot monopolize it any more and females move to other groups or together form a new group with a former bachelor male. Both situations seem to be fundamentally different from a multi-male group fission as described for baboons where individual in the daughter groups had been living together for a while before the split.

>> This is a fair concern regarding the comparability across species and sites. We’ve added text to clarify that many of these fissions occur when male takeovers divide a social group into multiple one-male groups. Our intention in referring to these taxa was to fold these examples into our broader definition of fission, in which we consider any demographic scenario in which one social group divides into multiple daughter groups. Also, the Sterck and Korstjens citation refers to a book chapter that discusses multiple cases where groups divided into new daughter groups and females are presented with the opportunity to join a new male or remain with a familiar male (including examples from not only colobines and gorillas but also chacma baboons, etc.). This broadly mirrors the scenario described for chacma baboons in the following sentence (L47-49).

Line 53: There is no reference provided for cases of aggressive herding. Does this occur in gorillas/langurs, in baboons or in both groups with male-initiated fissions?

>> We now clarify that this is pertaining to baboons in L49-50.

Line 56: I the connecting phrase likewise helpful here? You are sending the reader on a quest for what is similar between the two sentences without making it explicit.

>> We’ve removed this connecting phrase for clarity.

Line 63: What are THESE two fission patterns? The breaking kin bonds vs. not OR female vs. male initiated? Be explicit.

>> We now clarify our meaning in L56.

Line 86: What is the difference between dispersal patterns and fission outcomes? These terms have not been defined and are therefore ambiguous.

>> We agree that this was ambiguous, as they largely pertain to the same process, and have simplified the wording in L75-76.

Line 88: It is not clear why and how grooming network structure should shift prior to group fission, because the intro does not prime the reader.

>> We’ve added some text and provided citations demonstrating that social networks often change prior to fission in L38-39. We’ve also amended our research questions to make it clearer why we modeled grooming relationships across all of these periods.

Line 93: The topic of eviction and targeted aggression is not properly introduced. A reader may also expect to see targeted aggression to be contrasted with overall elevated aggression among females possibly resulting from increased feeding competition triggering female led fission.

>> We’ve simplified the text here to clarify the distinct possibilities and prime the readers for our analytical approach in L79-80.

Line 97: No predictions or background are provided about inter-group grooming except for mentioning that there is a lot of it.

>> We’ve added a sentence to make it clear that intergroup grooming is uncommon for baboons and clarify why we included these data in L70-73.

Line 186: The sequence of sections is a bit odd, because we read about how the data were analyzed for dominance relationships before we learn how data were acquired.

>> We’ve re-ordered these paragraphs for clarity.

Line 390: From the literature reviewed in the intro, it makes a lot of sense to ask whether parallel dispersal involved females with strong grooming relationships. So I understand why one would model grooming in period 1 as a function of final fission outcome, even if the causality is reversed here. What I do not understand is why this analysis is conflated with between group grooming which ought to be less frequent than within group interactions or the definition of group membership is off. Is it not self-evident that females that always stayed together had the strongest relationships in these models? I am looking across four periods that would include for everybody else 1-3 periods where partners did not co-reside which must reduce the opportunity to groom. It seems that the models of eventual outcome are just a different way of modelling co-residence in four steps instead of binary (the same goes for male-female relationships).

>> We now clarify in the Introduction that our aim in including these between-group ties was to examine whether the dispersing females engaged in “shopping” prior to their dispersal. In other words, did they start grooming with ENK females at higher rates before they decided to leave their natal group.

Line 242: Apart from the conceptual issue raised about Line 390ff, the paper would be much lighter if only the models with both predictors would be maintained. It is the best model for males and not worse than individual models for females. One way to assess how severely the covariation among predictors may have affected model outcomes is to use a frequentist glmm with the same model structure and investigate variation inflation factors. But see my comments re Line 390ff.

>> We agree in principle. However, due to the strong correlation between current group membership and eventual outcomes, solely presenting the models using both terms could inadvertently give the false impression that females do not groom most with the other females from their social groups. Thus, we opted to use this model comparison approach to avoid these issues and instead describe how these three models differentially capture observed variation in grooming relationship strengths.

Figure 2: The data in figure 2 (formerly Fig. 3) look very different between the two versions of the manuscript. Can the authors explain how all differences became so much more pronounced in the revised version when both the legends say the same which suggest the same data have been plotted?

>> Our apologies for the confusion. The original figure projected the posterior distributions conditional on grooming having occurred (i.e., only including grooming dyads that “passed” the hurdle). The updated visualizations thus are a truer reflection of the differences across these groups, as they allow “zero” values to emerge within the posterior.

Line 401: Like the dispersal outcome analyses in Model 1, the analysis of maternal relatedness and grooming across all periods does not seem very informative, because it just replicates co-residence effects. The interesting part is, whether maternal relatedness in period 1 predicted who ended up in different groups.

>> We agree that the role of maternal relatedness in fission outcomes is interesting, as this motivated our *post hoc* analysis focused on relatedness among dyads that did and did not split apart. That being said, we’re not entirely convinced that kinship effects within the grooming dataset merely reflect or absorb co-residence effects. Although maternal kin dyads were much more prevalent within groups than between groups, these still correspond to a small percentage of the total sample. Thus, including kinship as an additional covariate captures whether maternal kin groom each other above and beyond what would be expected given their co-residence patterns.

Line 423: An analysis of grooming vis-à-vis siring across all periods only makes sense, if males sire a lot of offspring outside their social group – otherwise the analysis will just reproduce co-residence patterns. In contrast, information on Yohan’s siring success with the dispersing versus non-dispersing PHG females makes a lot of sense.

>> Due to movement of males between the social groups, there were occasionally mother-sire dyads that were separated across the social groups. For instance, out of the 33 mother-sire dyads that were present during the final sampling period (i.e., the Fission period), 21 were co-resident and 12 were no longer co-resident. We now report these broader outcomes related to mother-sire dyads in L372-373.

Line 443: Is it not the grooming networks that became denser instead of the groups themselves.

>> We’ve reworded this for clarity in L381.

Did the grooming network analyses include subadult males? All other analyses seem to concern only fully adult individuals, but the laipikia_dispersal data frame has subadult males with network metrics.

>> Thanks for pointing this out. These age definitions are used by the broader UNBP project for other research purposes but were not the focus of analyses here. For simplicity, our analyses included data on all females above age 4 and all males above age 6 (as noted in L138-141), without reference to the specific life history or phenotypic criteria that would generally be used to designate them “subadult” or fully “adult.” To avoid confusion, we’ve now removed reference to the term ‘subadult’ in the data files and throughout the main text.

Line 521: Why is groups plural here? All 6 females that dispersed came from one group, right? Does it follow that only data from period 1 were analyzed to compare whether dispersing females were targeted for aggression which then led to dispersal in period 2 and 3? Or is it a question whether the females that went back to PHG had been targeted by ENK residents which triggered their (temporary) return home where they may have been targeted again leading to their permanent emigration? In other words: do these six females play the same social role across periods 1-3 and of not, does it make sense to model aggression received across all periods?

>> Good catch. We’ve changed plural “groups” to singular “group.” And, yes, those females played the role of “dispersers” across those sampling periods.

Line 559: It seems that the out-group grooming comparison is a bit unfair, because opportunity to groom outside the group increased in period 4 (fission) when the six dispersers gained many more out-group partners than they had in any other constellation.

>> That’s a fair point. We now mention this as a possible explanation for these temporal patterns in L521-522.

Line 563: It seems odd to say that fm out-group grooming was disproportionately directed from females to males when in fact all the many dyads involving a disperser male must have involved Yohan whereas a similar number of dyads in the fm category came from six dispersed females.

>> Apologies for this confusion. Yohan was not the only male to disperse during the study period. We now explicitly state that 4 out of 21 males from these social groups moved between the study groups at least once during the study in L519-520.

Line 586: Is the plural correct here, are there several males that had sired the dispersing females’ last offspring or is it all only about Yohan? If it is the former, because the events unfolded over such a long time, we need more details.

>> We’ve reworded this to clarify that we were focusing on Yohan. However, given this comment, we now extend our original descriptive statistics to demonstrate that mother-sire dyads often remained together even when the dyads including Yohan were excluded (L372-373).

**Reviewer #2**: Line 348: Seems like Yuhon did just find avoiding inbreeding before leaving PHG. In macaques, natal males from high-ranking matrilines can do very well without leaving. It could also be that Yuhon followed the first three dispersing females in order not to lose his access to preferred mates (ones he had already successfully bred with). Yes, Yohan reduces his inbreeding risk by dispersing, but the female-first dispersal scenario cannot be ruled out. This section is written as support for siring histories shaping female social decisions, but the same argument can be made for siring histories shaping male dispersal decisions in this case.

>> This is a good point. We’ve now revised these sentences to make it clearer that we can rule out these alternative explanations for Yohan’s dispersal. Yohan left PHG for the first time before any of the females had left. He then returned and left again along with several of the mothers of his infants.

Lines 355-356: “the dispersal of PHG females into ENK may have triggered social instability”. Maybe I don’t understand modularity well enough, but isn’t this just the result of the new arrivals not integrating well into the ENK. Does this really count as “social instability”? I would argue not, if the original ENK group members didn’t themselves become more modular in their associations. Maybe in connection with the increase in aggression does the “social instability” comment make more sense, but here it seems a bit out of place.

>> We agree that this was worded too strongly. We’ve modified the text in L377-380.

Line 358-360: Is this supposed to say either “female-male grooming” or "male-female grooming", instead of “83.8% of between-group grooming”?

>> Thank you for catching this error. We’ve corrected this in L386-387.

Line 443: Was the female that stayed in ENK not a member of the matriline? If so, it would be good information to state. An earlier sentence (line 301) mentions that she had kin ties with ENK.

>> We now note that she was not a member of the matriline in L316-317.

Figure S1.

- Describe what the colors/shading represent.

- “Co-residence” in a) is the same as “current membership” in b? If so, why different labels? It only introduces confusion.

- “co-residence likely facilitated past siring opportunities, while a prior siring history may compel males to have remained in that social group.” I am confused about this statement. If you change past siring history, then you can change an individual's decision about group membership (you removed a reason to stay). But, if you change an individual's current group membership, you don’t change the prior siring history. Please clarify.

>> Thank you for these suggestions. We’ve added a more informative legend to clarify the colors and have standardized the labels across the two DAGs.

The authors don’t mention whether they checked trace plots to see if each chain was ‘behaving well’. Authors don’t mention if they ran prior predictive checks (typically in brms: pp_check()).

>> We’ve added this information in L307-311.

**Reviewer #3**: The editor asked me only to review the statistical methods. This section of the manuscript has considerably improved, and the applied statistical tests are now much clearer explained. I still have a few comments:

L181: Please specify: which contain many zeroes in the response variable

>> We now clarify this in L182-184.

L186-190: I would suggest moving this explanation to the results section.

>> We’ve modified the text to improve readability in 326-327 and 358-359.

L194: Maybe “dyadic” instead of “interaction”?

>> We’ve made this edit in L194.

L200: Mention the response variable you used (grooming duration measured in minutes?)

>> We now clarify this in L186.

L219-220: Please add how many dyads were omitted.

>> We’ve added this information in L235-236.

L231-243: This is an interesting approach to overcome the issue with differences in sampling effort. However, I am unsure if the posterior predictions of the two models (female-female, female-male) can be pooled. The models were run with a different set of predictors (mat relatedness and rank difference for same sex, male rank and recent common offspring for different sex). They might capture different relationships between the predictors and the response. Pooling these predictions essentially assumes that the effects of the main predictor (co-residency, eventual outcome) are directly comparable across these two models, despite the different context set by the control predictors. Please elaborate on this.

>> That is a fair concern. Our intention in pooling these data was to ensure that the networks generated using posterior estimates reflected shifts in the density and modularity of both female-female and female-male grooming relationships combined (and not simply the former). While these data derive from two models drawn from two distinct dataframes, our intention was to examine the combined contributions of these relationship types. However, to ensure that the posterior predictions from female-female and female-male dyads stem from more congruent models, we’ve modified this analysis to draw posterior predictions from Models 1c and 1f (i.e., the models including both current group membership and eventual outcome, and not merely the best performing model).

L247-248 and L276-280: Please report the percentage of zeroes in the response variable here as well.

>> We now include this information in L294-295.

L290-294: The authors report that they ran the models in brms with 4 chains with 2,000 iterations each, which is the default setting for brms. Of these 2,000 iterations per chain, by default brms runs 1,000 iterations each to warm-up and 1,000 iterations to sample. As the authors did not specify the number of warm-up and sampling iterations used, I assume they applied default settings. However, in the supplementary, they report bulk and tail ESS of well over 4,000 for most parameters. To my understanding, such high ESS values are only possible when >> 2,000 iterations were used. Please clarify which settings you used for which model.

>> We’ve clarified these settings and corrected this error in L307-311.

L292: Why was 500 used as a threshold for bulk and tail ESS? The default of brms is ~ 1,000 to consider an estimate stable. Also, your reported results exceed 500 by far. Please clarify.

>> Same as above.

L293-294, L186-190: Even though the authors apply Bayesian statistics, they rely on a certain threshold to differentiate between biologically meaningful and non-meaningful results. However, it is advised not to stick to arbitrarily chosen thresholds to assess biological relevance in a Bayesian approach (even though 89% is widely used, its initial choice was arbitrary; McElreath 2020, Statistical Rethinking). E.g. the authors report that rank differences did not predict grooming relationships (L319-320), even though the zero is very close to the upper boundary of the CI. Based on these numbers, I would argue that there might be a pattern, just not a strong one (i.e., a “trend” in frequentist terms), although one would need to see the whole posterior distribution for proper interpretation. Hence, I think the manuscript would benefit a lot from adding visualisations of the posterior distributions of model estimates. This would make it much easier to compare the position and breadth of distributions among each other and get a feeling for the certainty and biological meaning of the estimates.

>> Thank you for these suggestions. We’ve now softened our language regarding the marginal effect of rank on grooming relationships and included visualizations of the posterior distributions of our best-performing models in the supplementary materials.
